# Exosomes from BM-MSCs promote acute myeloid leukemia cell proliferation, invasion and chemoresistance via upregulation of S100A4

**DOI:** 10.1186/s40164-021-00220-7

**Published:** 2021-03-31

**Authors:** Tianxin Lyu, Yinuo Wang, Ding Li, Hui Yang, Bin Qin, Wenli Zhang, Zhiyue Li, Cheng Cheng, Binglei Zhang, Rongqun Guo, Yongping Song

**Affiliations:** 1 Department of Hematology, Affiliated Cancer Hospital of Zhengzhou University, Zhengzhou, 450008 China; 2 Academy of Medical Sciences, Zhengzhou University, Zhengzhou, 450052 China; 3 Translational Cancer Research Center, Peking University First Hospital, Beijing, 100034 China; 4 Department of Pharmacy, Affiliated Cancer Hospital of Zhengzhou University, Zhengzhou, 450008 China; 5 Department of Hematology, The First Affiliated Hospital of Zhengzhou University, Zhengzhou, 450052 China

**Keywords:** Acute myeloid leukemia, Exosome, Mesenchymal stem cells, Invasion, Chemoresistance

## Abstract

**Background:**

BM-MSCs play an important role in cancer development through the release of cytokines or exosomes. Studies have shown that extracellular exosomes derived from BM-MSCs are a key pro-invasive factor. However, how BM-MSC-exos influence AML cell proliferation, invasion and chemoresistance remains poorly understood.

**Methods:**

We isolated exosomes from BM-MSCs and used electron microscopy, particle size separation and western blots to identify the exosomes. The invasion of leukemia cells was observed with a transwell assay. The stemness traits and chemoresistance of the leukemia cells were detected by FCM, colony formation and CCK-8 assays. TCGA database was used to investigate the prognostic relevance of *S100A4* and its potential role in AML.

**Results:**

In this study, we found that BM-MSC-exos increased the metastatic potential, maintained the stemness and contributed to the chemoresistance of leukemia cells. Mechanistically, BM-MSC-exos promoted the proliferation, invasion and chemoresistance of leukemia cells via upregulation of S100A4. Downregulating S100A4 clearly suppressed the proliferation, invasion, and chemoresistance of leukemia cells after treatment with BM-MSC-exos. Bioinformatic analysis with data in TCGA database showed that S100A4 was associated with poor prognosis in AML patients, and functional enrichment revealed its involvement in the processes of cell–cell adhesion and cytokine regulation.

**Conclusions:**

S100A4 is vital in the BM-MSC-exo-driven proliferation, invasion and chemoresistance of leukemia cells and may serve as a potential target for leukemia therapy.

## Introduction

Acute myeloid leukemia (AML) is a clinically heterogeneous hematologic malignancy characterized by an elevated level of differentiation impairment and by the hyperproliferation of nonlymphoid progenitor cells in the bone marrow or peripheral blood. From 1990 to 2017, the incidence of AML gradually increased worldwide, and the burden of AML became heavier [1, 2]. The genomic heterogeneity and characteristics of AML patients impact their clinical outcomes [3, 4]. With the appearance of new drugs and therapies, 35% to 40% of patients younger than 60 years of age and 5% to 15% of those older than 60 years now have curable disease [5]. However, the outcomes of some patients who are older or unable to receive intensive therapy remain dismal [6]. Leukemia originates from and is maintained by leukemic stem cells (LSCs), which have many similarities to hematopoietic stem cells (HSCs), including self-renewal capacity and multidifferentiation features [7]. Previous studies have revealed that LSCs can produce leukemic progenitor cells, which differentiate into primitive leukemic cells that are blocked at different stages of the cell cycle [8]. It is difficult to kill these cells via conventional chemotherapy since most of them are in the G0 stage; thus, recurrence and chemotherapy resistance often occur [9]. Therefore, more studies on LSCs are necessary and important for the research and development of leukemia treatment options.

In recent years, an increasing number of studies have shown that stromal cells, working as tumor microenvironment components, play a vital role in tumor cell proliferation and drug resistance [10]. Bone marrow mesenchymal stem cells (BM-MSCs) are important components of the bone marrow microenvironment, and their interaction with tumor cells can promote tumor formation and progression [11]. MSCs physically contact with adjacent tumor cells and can secrete cytokines. Those cytokines can trigger intercellular signaling, which directly affects the dynamics and fate of tumor cells [12]. In addition, evidence indicates that there are some special transmission systems in which exosomes work as carriers in MSCs [13].

Exosomes are composed of lipid bilayer vesicles containing various bioactive molecules, including DNA, microRNA (miRNA), proteins, and lipids [14–16]. Exosomes secreted by cells act as important mediators between tumor cells and stromal cells by transferring information from the bone marrow microenvironment and shaping the microenvironment [17–19]. Hence, exosomes derived from BM-MSCs (BM-MSC-exos) play a significant role in cancer development [20, 21]. Studies have shown that [22] BM-MSC-exos increase the population of cancer stem cells (CSCs) in colon cancer cells by transferring miR-142-3p. However, the effect of BM-MSC-exos on LSC-like traits is still unclear. In addition to invasion, drug resistance is a major problem causing relapse or death during clinical cancer treatment [23]. Emerging evidence has suggested that BM-MSC-exos induce the resistance of cancer cells to different types of drugs [24]. However, the exact mechanism has not been well elucidated.

S100A4, a typical member of the S100 family of calcium-binding proteins, has both intracellular and extracellular functions involving the regulation of various biological processes, such as angiogenesis, cell growth, motility, differentiation, apoptosis, and invasion [25–29]. S100A4 has been reported to be highly expressed in various cancers and is usually associated with a poor prognosis [30–33]. Knockdown of *S100A4* in tumor cells not only strongly impacts their survival but also reduces the stem cell-like phenotype of the tumors [33, 34]. In addition, *S100A4* knockdown results in an increase in the sensitivity of pancreatic ductal adenocarcinoma cell lines to gemcitabine treatment, which is coupled with an increase in apoptosis and cell cycle arrest [35].

In this study, we found that BM-MSC-exos increased the population of LSCs, enhanced the chemoresistance of leukemic cells, and promoted the release of leukemic cells into peripheral blood. Mechanistically, this function was related to the upregulation of S100A4 from BM-MSC-exos into leukemic cells. Altogether, we demonstrated that exosomes upregulated the expression of S100A4 and that this played an important role in the proliferation, invasion and chemoresistance of leukemic cells.

## Methods

### Isolation of human MSCs

A bone marrow aspirate totaling 50 ml was obtained from the posterior superior iliac spine of 10 healthy donors (mean age 41.7 years, range 28 to 55 years; 10 males) undergoing bone marrow puncture. All participants provided written informed consent. The study followed the ethical guidelines and was approved by the Ethics Committee of Affiliated Cancer Hospital of Zhengzhou University (approval no. 2020239). Then, the marrow was mixed gently with heparin to prevent coagulation. After dilution with phosphate-buffered saline (PBS), the mixture was added to a tube containing the same amount of lymphocyte separation solution and centrifuged at 2000 rpm for 20 min. Then, the mononuclear cells in the white membrane were collected, washed and centrifuged twice with PBS. The mononuclear cells were cultured in culture medium (NutriStem MSC XF Basal Medium, Biological Industries, 05–200-1A; NutriStem MSC XF Supplement, Biological Industries, 05-201-1U) and incubated in a 5% CO_2_, 37 °C incubator. After incubation for 48 h, the media was refreshed, and the nonadherent cells were discarded with the exchanged culture medium. The remaining adherent cells were MSCs.

### Exosome isolation

BM-MSCs were cultured in fetal bovine serum (FBS)-free culture medium, and the culture medium was collected by centrifugation at 5000 rpm for 10 min, followed by filtration through a 0.22 μm filter. Then, we added moderate EP solution (Beibei Biotechnology Co., Ltd., China) to the supernatant and mixed gently. Then, after incubation for 1 h at 0–4 °C, the mixed solution was centrifugated at 12,000 rpm, 4 °C for 15 min. The supernatant was removed and the obtained precipitates contained the exosomes. Finally, after resuspension in 500 ul of PS buffer (Beibei Biotechnology Co., Ltd., China), the exosomes were stored at − 80 °C.

### Electron microscopy

Exosomes were suspended in 50–100 μl of 2%. A 5 μl exosome suspension was dropped onto the copper mesh and kept at room temperature for 20 min, followed by washing with PBS 3 times for 5 min. Then, the droplet was fixed with 1% glutaraldehyde for 5 min and washed with ddH_2_O 10 times for 2 min. After that, we added 4% uranium dioxo acetate for 5 min for negative staining and used filter paper to absorb any residual liquid. The sample was dried at room temperature. Finally, the grids were observed at 120 kV with a transmission electron microscope (TECNAI G2, FEI company, USA).

### Nanoparticle tracking

For nanoparticle tracking analysis (NTA), the sizes of the exosomes were characterized by dynamic light scattering (Nanosight NS300, Malvern Instruments, Malvern, UK) according to manufacturer’s instructions. Exosomes were diluted in pure water to obtain 20–40 particles/view.

### Western blot analysis

Equal amounts of total protein were separated by SDS-PAGE and transferred to PVDF membranes (Merck Millipore, Billerica, MA, USA). The membranes were incubated with 5% skim milk for 1 h at 37 °C, washed with TBST, and then probed for 2 h at 37 °C with antibodies targeting CD63 (1:5000 dilution, Biolegend, CY5253, USA), TSG101 (1:5000 dilution, Biolegend, CY5985, USA), S100A4 (1:1000 dilution, Abcam, ab124805, USA) and β-actin (1:3000 dilution, Abways, AB0035, China). Then, the membranes were incubated with HRP-labeled anti-rabbit IgG (1:5000 dilution, Abways, AB0101, China) for 1 h at 37 °C. After washing, the coloration was detected with 3,3-diaminobenzidine tetrahydrochloride (DAB; Sigma-Aldrich, St. Louis, MO, USA) or using an enhanced chemiluminescent kit (New Cell & Molecular Biotech Co., Ltd, Suzhou, China).

### Cell culture and treatment

Human AML lines THP-1 and KASUMI-1 were obtained from the Tianjin Institute of Blood, Chinese Academy of Medical Sciences. Leukemia cell lines were validated by the short tandem repeat method. All the cells were maintained in RPMI-1640 medium with 10% FBS (GIBCO, 10099141C) and 1% antibiotic–antimycotic solution (Life Technologies, NY). For exosome treatment, leukemia cells were treated with 50 μg/ml BM-MSC-exos or PBS.

### Flow cytometry (FCM) analysis

Staining was performed with CD123-PE/Cyanine7 antibody (Biolegend, Clone 6H6), CD34-PE antibody (Biolegend, Clone 581), CD45-FITC antibody (Biolegend, Clone H130), HLA-DR-PE/Cyanine7 antibody (Biolegend, Clone L234), CD44-FITC antibody (Biolegend, Clone BJ18), CD73-Brilliant Violet 421 antibody (Biolegend, Clone AD2), CD90-APC antibody (Biolegend, Clone 5E10), and CD105-PE antibody (Biolegend, Clone SN6h) according to the manufacturer’s instructions. FCM analysis was subsequently performed using a BD LSRFortessa cytometer.

### Cell Counting Kit-8 (CCK-8) assay

In vitro drug resistance was assessed by the CCK-8 assay. Leukemia cells were treated with exosomes or PBS and replated into a 96-well plate (20,000 cells per well). (Ara-C) was added to the cells at different concentrations for 48 h, the assays were performed by incubating the cells in each well with 20 μl CCK-8 substrate for 3 h, and the optical density (OD) values were read at a wavelength of 450 nm.

### Colony formation assay

In the colony formation assay, cells were diluted with RPMI-1640 culture medium containing 2% FBS. Then, methylcellulose was added to the cell suspension at a ratio of 1:10; the mixture was shaken fully and incubated for 20 min. The cells were then seeded in a 24-well plate (200 cells per well). After 14 days, colonies were photographed with an inverted fluorescence microscope. For exosome treatment, leukemia cells (KASUMI-1 and THP-1) were treated with 50 μg/ml BM-MSC-exos or PBS as the control.

### Cell migration and invasion assay

Transwell cell invasion assays were performed by using Boyden chambers with polycarbonate nucleopore membranes (6.5 mm in diameter, 8-µm pore size) coated with matrigel. The polycarbonate nucleopore membrane without matrigel was used for the transwell cell migration assays. Then, 200 μl serum-free RPMI-1640 medium containing 1 × 10^5^ cells was placed in the upper part of each chamber, and the lower compartment was filled with 600 µl RPMI-1640 culture medium containing 10% serum previously. For exosome treatment, leukemia cells (THP-1 and KASUMI-1) were treated with 50 μg/ml BM-MSC-exos or PBS.

### Quantitative real-time PCR assays

mRNA was isolated with TRIzol reagent (Invitrogen) and reverse transcribed (R323-01, HiScript III RT SuperMix for qPCR (+ gDNA wiper), Nanjing Vazyme Co., Ltd.). cDNA was amplified by reverse transcriptase-PCR. Transcription assays were used to quantify the transcription levels of the following mRNAs: *OCT4*, *BMI1*, *KLF4*, *NANOG*, *SOX2* and *GAPDH*. Quantitative PCR (Q711-02, ChamQ Universal SYBR qPCR Master Mix, Nanjing Vazyme Co., Ltd.) was conducted in triplicate at 95 °C for 30 s, followed by 40 cycles of 95 °C for 10 s and 60 °C for 30 s (7500 Fast Real-Time PCR System; Applied Biosystems). Cycle threshold values were normalized to those of an internal control (*GAPDH* for the mRNA assays). The gene expression levels were calculated using the 2^−△△CT^ method, and the levels of mRNA were normalized to that of the adopted internal control (denoted as relative expression).

The primers used in the qPCR assays are listed as follows.

**Table:** 

Gene	Forward	Reverse
*OCT4*	AGCCCTCATTTCACCAGGCC	CCCCCACAGAACTCATACGG
*BMI1*	AGCAGCAATGACTGTGATGC	CAGTCTCAGGTATCAACCAG
*KLF4*	CAGCTTCACCTATCCGATCCG	GACTCCCTGCCATAGAGGAGG
*NANOG*	TCCAGGATTTTAACGTTCTGCT	TTCTTGCATCTGCTGGAGGC
*SOX2*	ACACCAATCCCATCCACACT	GCAAACTTCCTGCAAAGCTC
*GAPDH*	GCACCGTCAAGGCTGAGAAC	TGGTGAAGACGCCAGTGGA
*S100A4*	GATGAGCAACTTGGACAGCAA	CTGGGCTGCTTATCTGGGAAG

### RNA interference experiment

Two *S100A4* small interfering RNAs (siRNAs) were purchased from Sangon Biotech (Shanghai, China). To knockdown *S100A4* expression, 50 nM siRNA against *S100A4* was transfected into AML cells using Lipofectamine 3000 (Invitrogen, L3000015), as suggested by the manufacturer. The sequences of the two *S100A4* siRNAs are listed as follows.

**Table:** 

	Sense	Antisense
Human negative control	UUCUCCGAACGUGUCACGUTT	ACGUGACACGUUCGGAGAATT
Human *S100A4*-1	UGUCCACCUUCCACAAGUATT	UACUUGUGGAAGGUGGACATT
Human *S100A4*-2	UCCAGAAGCUGAUGAGCAATT	UUGCUCAUCAGCUUCUGGATT

### Data accession from The Cancer Genome Atlas (TCGA) database

RNA-seq and clinical information on AML cases were retrospectively obtained from TCGA database.

### Identification of differentially expressed genes (DEGs)

The “limma” R package was used to identify the DEGs between the *S100A4* high and low groups. Genes with a FDR < 0.05 and a |log2 Fold Change (log2FC)|≥ 0.5 were considered differentially expressed. The online database Gene Expression Profiling Interactive Analysis 2 (GEPIA2; http://gepia2.cancer-pku.cn/#index) was used to validate the expression of *S100A4* between tumor and normal tissues.

### Survival analysis

Univariate cox regression and multivariate regression were carried out with the “survival” R package. Kaplan Meier (KM) curves were used to evaluate the prognosis as it related to *S100A4* using the “survminer” R packages.

### Gene Ontology (GO) and Kyoto Encyclopedia of Genes and Genomes (KEGG) pathway enrichment analyses

GO and KEGG analyses were performed using the “clusterProfiler” R package based on the DEGs between patients with high and low *S100A4* expression. The GO results contained three categories: biological processes (BP), cellular components (CC) and molecular functions (MF). Enriched GO terms and KEGG pathways were determined according to the adjusted critical criterion of P < 0.05.

### Statistical analyses

In the study, all data are shown as the mean ± standard deviation (SD). Shapiro–Wilk’s test and Levene’s test were used to check the data normality and homogeneity, and one-way ANOVA and t-test were used to analyze the difference between various experimental groups. The threshold for statistical significance was P < 0.05.

### Study approval

The use of normal donor bone marrow specimens was evaluated and approved by the Ethical Committee of Affiliated Cancer Hospital of Zhengzhou University (approval no. 2020239), and written informed consent was obtained from all participants or their appropriate surrogates.

## Results

### Isolation and identification of BM-MSCs and BM-MSC-exos

**Fig. 1:**
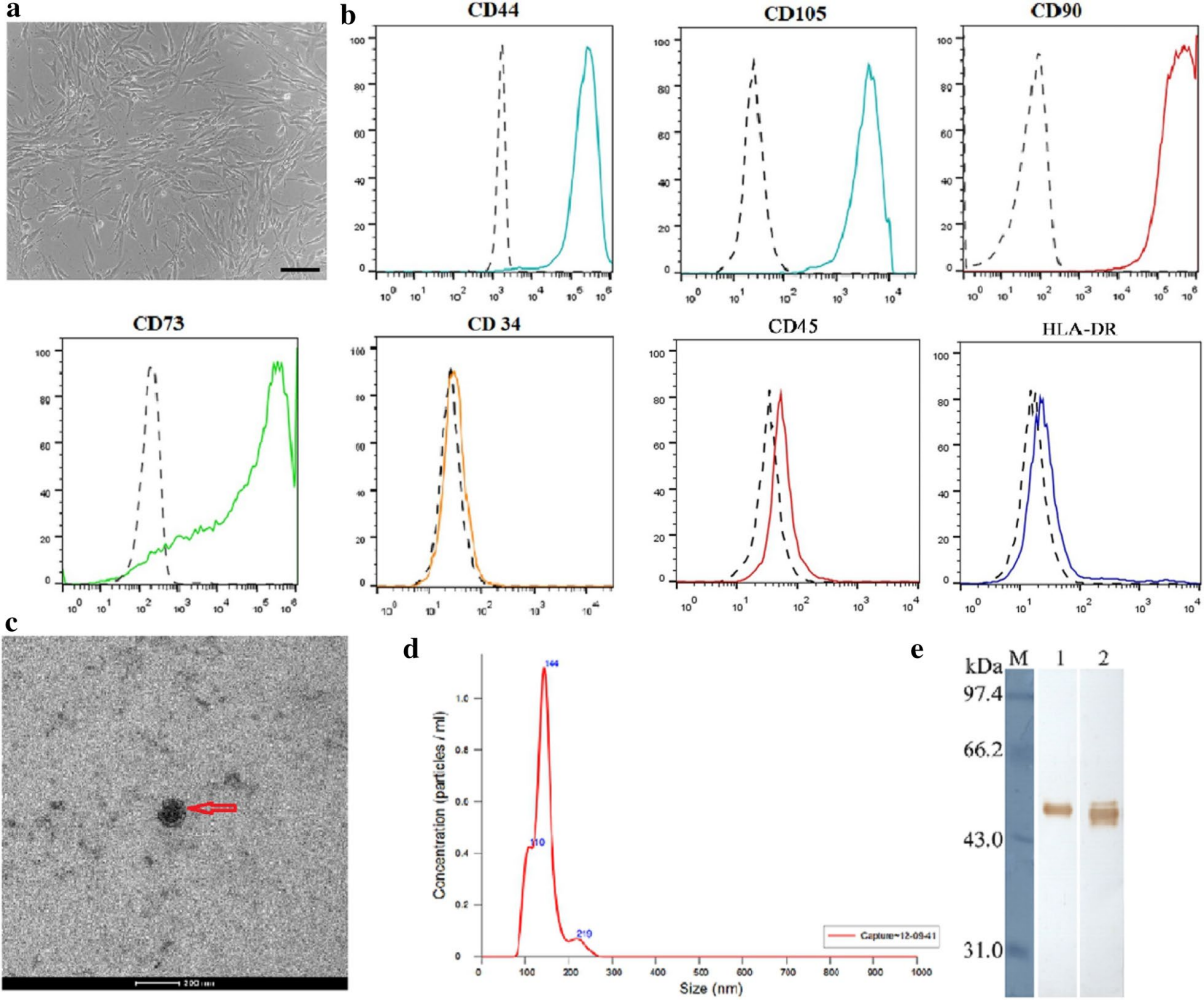
Characteristics of BM-MSCs and BM-MSC-exos. **a** Representative morphology of BM-MSCs. Magnification: × 40, scale bar: 500 µm. **b** FCM analysis showed that BM-MSCs were positive for CD44, CD73, CD90, and CD105, and negative for CD34, CD45, and HLA-DR. **c** Electron microscopy of BM-MSC-exos, showing small vesicles of 128.3 × 100 nm; scale bar: 200 nm. **d** Size distribution of BM-MSC-exos by NTA. **e** Western blot analysis showed the expression of TSG101 (1) and CD63 (2) in BM-MSC-exos

To obtain exosomes derived from BM-MSCs, we first purified human BM-MSCs from bone marrow cells donated by healthy donors. The cells showed a homogenous fibroblastic morphology (Fig. 1a) and expressed uniform surface markers; they were positive for CD44, CD73, CD90, and CD105, and negative for CD34, CD45, and HLA-DR after 4–6 passages (Fig. 1b). The morphology of exosomes was physically homogeneous and is shown in Fig. 1c. Using a particle size analyzer, we demonstrated that the width of exosomes ranged from 62.5 to 295.5 nm (Fig. 1d). To further identify the BM-MSC-exos, we performed western blotting to detect the expression of CD63 and TSG101, which are commonly considered specific exosomal markers [36]. As shown in Fig. 1e, both of the proteins were highly expressed in the BM-MSC-exos we isolated.

### BM-MSC-exos promoted the metastatic potential of leukemia cells

**Fig. 2:**
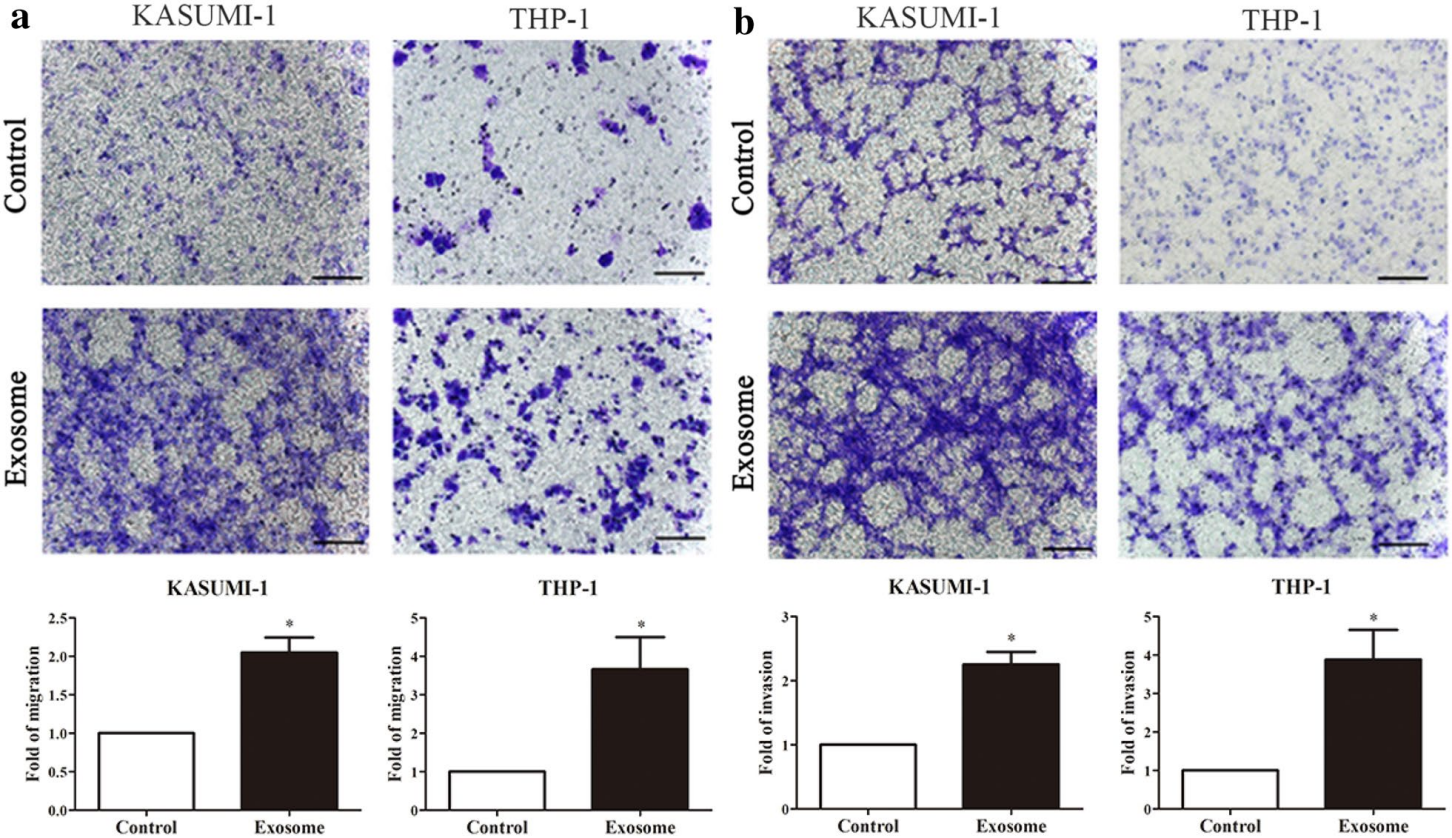
BM-MSC-exos promote the metastasis of leukemia cells. Cell migration (**a**) and invasion (**b**) of leukemia cells cultured with or without exosomes normalized to the migration index of cells cultured without exosomes; scale bar: 100 μm. Data are the means ± SD (n = 3; *P < 0.05; cells cultured with BM-MSC-exos vs. cells cultured with PBS; Student’s t-test)

To identify whether BM-MSC-exos could promote the metastasis of leukemia cells, leukemia cells (KASUMI-1, and THP-1) were treated with exosomes derived from conditioned medium of BM-MSCs. We performed cell migration and invasion assays. The cell migration assays showed that after treatment with BM-MSC-exos, leukemia cells exhibited significantly increased cell migration (P < 0.05) (Fig. 2a). Similar results were observed in the cell invasion assay (P < 0.05) (Fig. 2b). These findings suggest that BM-MSC-exos play a critical role in promoting the metastatic potential of leukemia cells.

### BM-MSC-exos increased the stemness and chemoresistance of leukemia cells

**Fig. 3:**
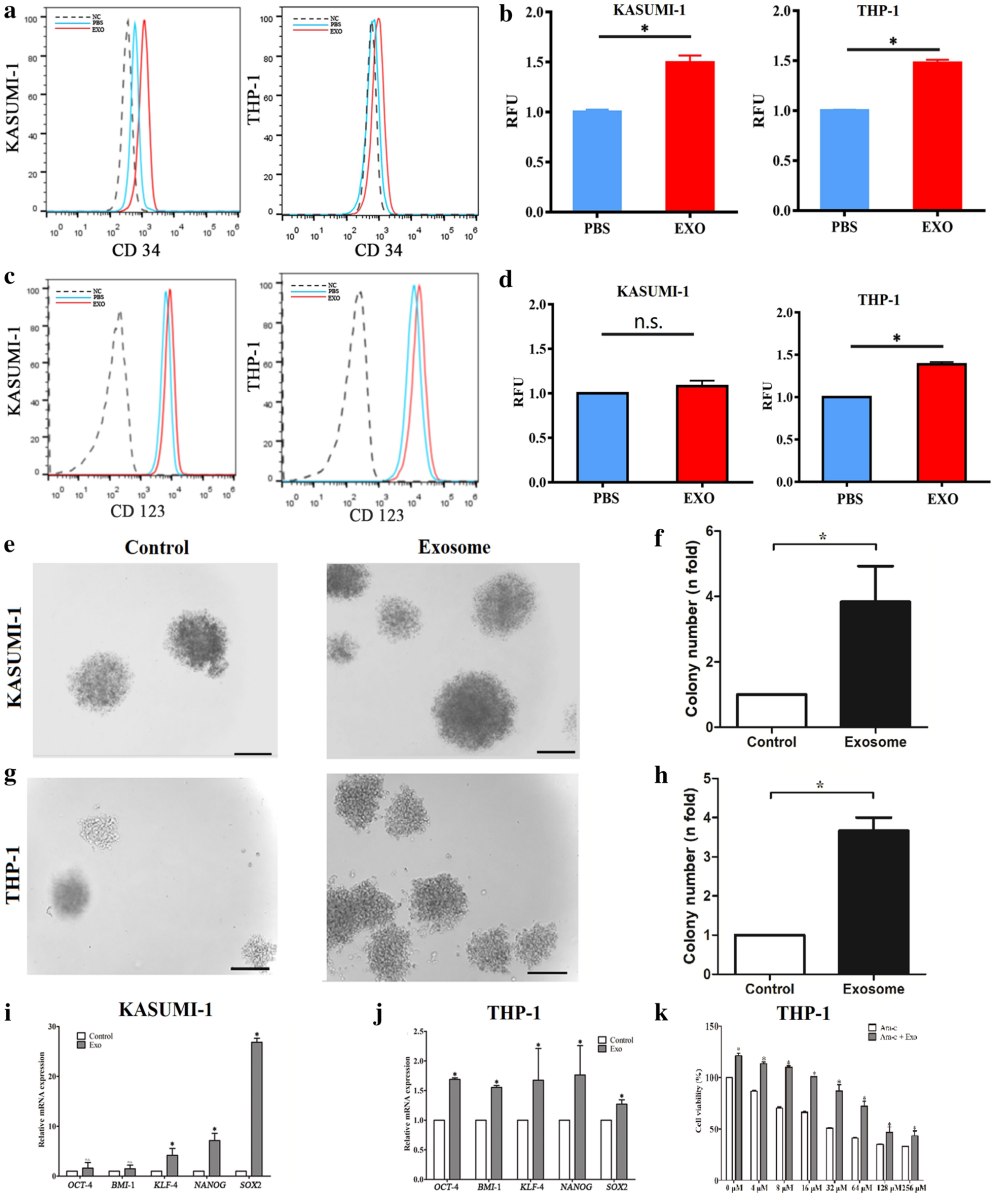
BM-MSC-exos increased the stemness and chemoresistance of leukemia cells. **a-d** Leukemia cells were cocultured with BM-MSC-exos or treated with PBS, and the CD34 (a,b) and CD123 (c,d) cell populations were determined. **e–h** Colony formation assays showed that the cell colony forming ability was enhanced upon coculture with BM-MSC-exos. **i**, **j** The expression of stemness genes (OCT4, BMI1, KLF4, NANOG and SOX2) in leukemia cells (KASUMI-1 (i) and THP-1 (j)) was increased upon treatment with BM-MSC-exos. **k** Drug resistance assay. Leukemia cells (THP-1) were treated with different concentrations of Ara-C with or without BM-MSC-exos. Data are the means ± SD (n = 3; *P < 0.05; n.s., not significant; cells cultured with BM-MSC-exos vs. cells cultured with PBS; Student’s t-test)

FCM was performed to test whether BM-MSC-exos could promote the stemness of AML cells, and the results revealed that exosome treatment increased the expression levels of CD34 (Fig. 3a) and CD123 (Fig. 3c) in leukemia cells (P < 0.05). The mean fluorescence of CD34 (Fig. 3b) and CD123 (Fig. 3d) in exosome-treated cells was approximately two times that of the control cells (P < 0.05); only the expression of CD123 by KASUMI-1 cells after exosome treatment was not significantly different from that of the control group. We also performed a colony forming assay to further measure the proliferative ability of leukemia cells treated with BM-MSC-exos. The results showed that the number of colonies formed by cells treated with exosomes was significantly higher than by cells treated with PBS (Fig. 3e, g), and there was a statistical difference between the two groups (Fig. 3f, h). In addition, the expression of stemness genes (*OCT4*, *BMI1*, *KLF4*, *NANOG*, and *SOX2*) was assessed by qPCR, and the results showed that these markers were upregulated in cells treated with exosomes compared with those treated with PBS (Fig. 3i, j). Furthermore, the leukemia cell line THP-1 was treated with different concentrations of Ara-C with or without BM-MSC-exos and cell viability was observed; the results showed that more cells survived Ara-C treatment when they were cotreated with BM-MSC-exos (Fig. 3k). Taken together, these data indicated that BM-MSC-exos increased the stemness and chemoresistance of leukemia cells.

### BM-MSC-exos promoted leukemia cell migration and invasion via upregulation of S100A4

**Fig. 4:**
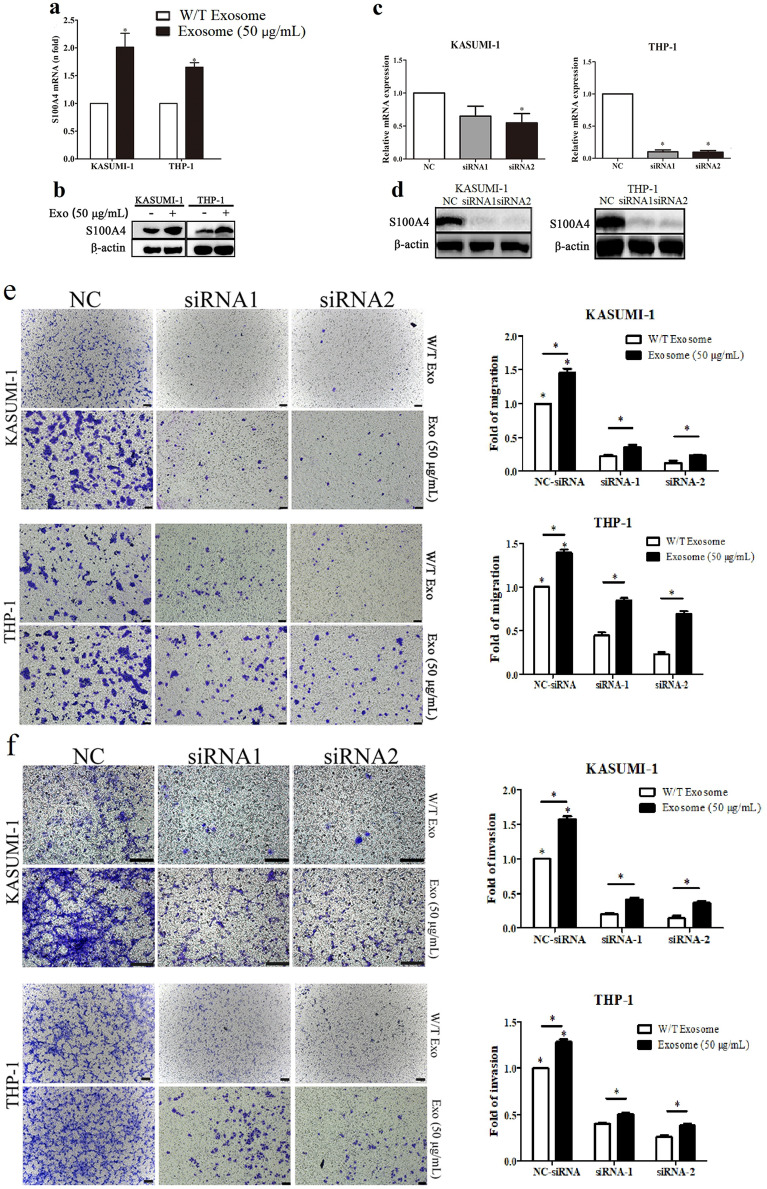
BM-MSC-exos promoted leukemia cell invasion via upregulation of S100A4. **a** BM-MSC-exo treatment significantly upregulated S100A4 expression in leukemia cells. **b** Western blotting verified the upregulation of the S100A4 protein upon exosome treatment. The knockdown efficiency was evaluated by both qRT-PCR (**c**) and western blot (**d**) analyses. S100A4 knockdown attenuated the migration (**e**) and invasion (**f**) of leukemia cells induced by BM-MSC-exos. Scale bar for the migration groups (KASUMI-1 and THP-1): 200 μm. Scale bar for the invasion group (KASUMI-1): 100 μm. Scale bar for the invasion group (THP-1): 200 μm. Data are the means ± SD (n = 3; *P < 0.05; n.s., not significant)

S100A4 is an important effector in tumor migration and invasion processes [30]. Previous studies have shown that increased expression of S100A4 affects proliferation and drug resistance in leukemia [33, 37]. Moreover, studies have shown that BM-MSC-exos containing cargo play an important role in leukemia progression [38–42]. Therefore, we wanted to determine whether BM-MSC-exos could induce the expression of *S100A4* to affect AML progression. We first verified the upregulation of *S100A4* mRNA in leukemia cells treated with BM-MSC-exos by qPCR (Fig. 4a) and S100A4 protein abundance by western blotting (Fig. 4b). These results suggested S100A4 as a possible target in the clinical treatment of AML and that it may be regulated by exosomes.

To examine the effect of *S100A4* deficiency on migration and invasion, we employed *S100A4* RNA interference in leukemia cells (Fig. 4c, d). The effect of *S100A4* on the migration and invasion of leukemia cells was observed with transwell migration and invasion assays. As shown in Fig. 4e, fewer cells migrated in the *S100A4* knockdown group than in the negative control (NC) group (P < 0.05). Moreover, while more leukemia cells migrated after treatment with BM-MSC-exos than in the NC group (P < 0.05), the migration ability of leukemia cells with *S100A4* knockdown and BM-MSC-exos treatment was weaker than that of leukemia cells with BM-MSC-exos treatment alone (P < 0.05). Similarly, after *S100A4* knockdown, fewer leukemia cells invaded the membrane (Fig. 4f). These data indicated that *S100A4* knockdown suppressed the migration and invasion of leukemia cells induced by BM-MSC-exos.

### *S100A4* knockdown decreased the proliferation and chemoresistance of leukemia cells after treatment with BM-MSC-exos

**Fig. 5:**
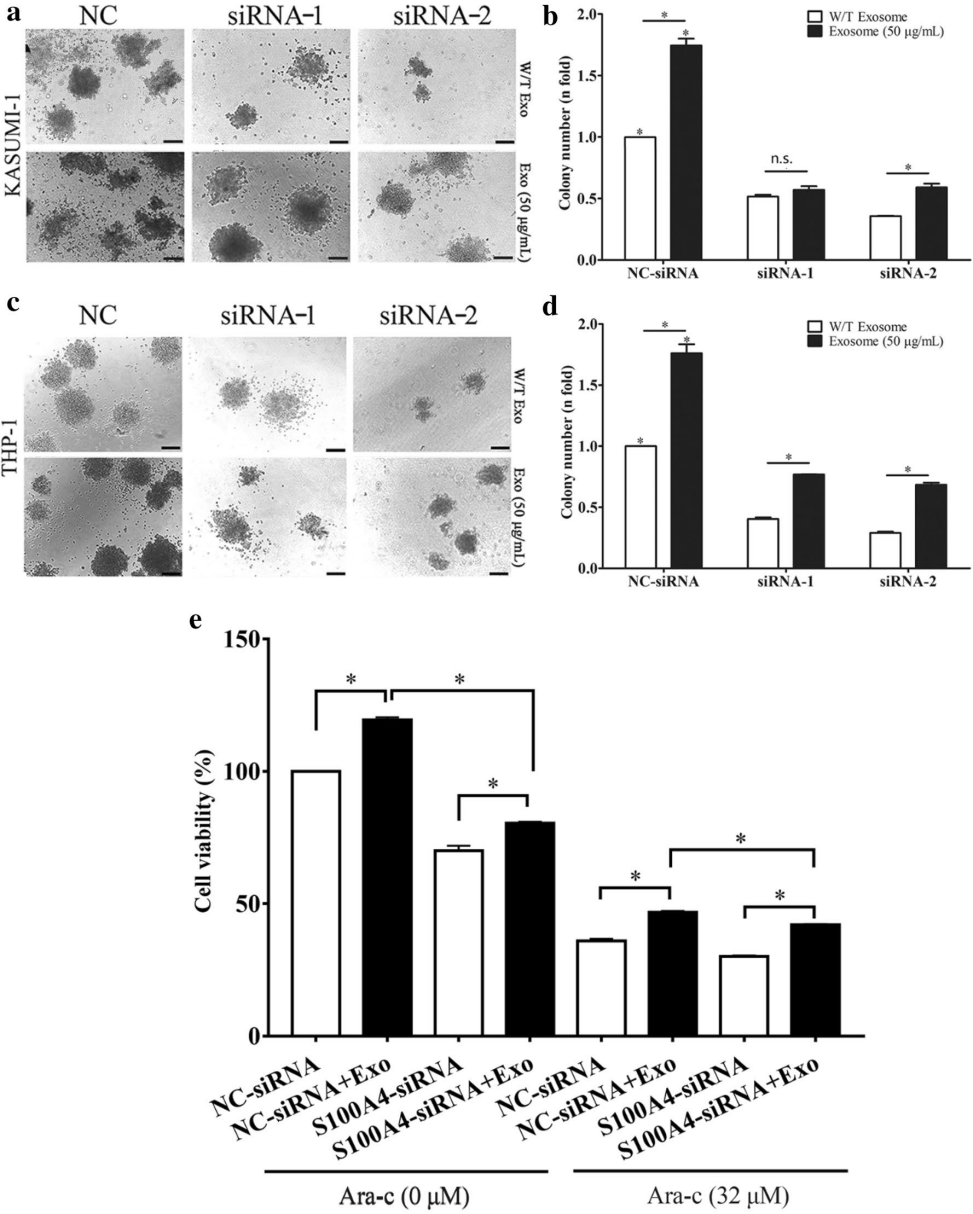
*S100A4* knockdown decreased the proliferation and chemoresistance of leukemia cells after treatment with BM-MSC-exos. **a**, **b** *S100A4* knockdown decreased the colony formation of KASUMI-1 cells induced by BM-MSC-exos. **c**, **d** *S100A4* knockdown reduced the colony formation of THP-1 cells induced by BM-MSC-exos. Scale bar for KASUMI-1 and THP-1: 100 μm. **e** CCK-8 assay was performed to observe the survival of THP-1 cells after Ara-C (32 μM) treatment with NC-siRNA, NC-siRNA + Exos, S100A4-siRNA, and S100A4-siRNA + Exos (*P < 0.05; n.s., not significant)

To examine the effect of *S100A4* deficiency on the proliferation and chemoresistance of leukemia cells, we employed *S100A4* RNA interference in leukemia cells treated with PBS or BM-MSC-exos. Colony forming assays were performed to detect the proliferative potential of leukemia cells. As shown in Fig. 5a, b, we found that there were significantly fewer colonies in the *S100A4* knockdown group than in the NC group (P < 0.05). In addition, BM-MSC-exo-treated leukemia cells formed significantly more colonies than PBS-treated leukemia cells (P < 0.05). As shown in Fig. 5c, d, we also found that BM-MSC-exo-treated leukemia cells formed significantly more colonies than leukemia cells treated with BM-MSC-exos and *S100A4*-siRNA (P < 0.05). Our previous results showed that more cells survived Ara-C treatment (32 µM) when they were co-treated with BM-MSC-exos. A CCK-8 assay was performed to observe the survival of leukemia cells in the absence of *S100A4*. As shown in Fig. 5e, similar to our previous data, more cells survived after treatment with BM-MSC-exos, and there was a significant difference in cell viability between the BM-MSC-exo-treated group and the group without BM-MSC-exos treatment. We observed an obvious decreasing trend after treatment with BM-MSC-exos and *S100A4*-siRNA, which may indicate that the increase in survival caused by BM-MSC-exos was partly attenuated by *S100A4* knockdown. Taken together, these data indicate that *S100A4* knockdown may decrease the proliferation and rescue the chemoresistance induced by BM-MSC-exos.

### S100A4 is a risk factor for AML and is manly associated with cell adherence and cytokine regulation

**Fig. 6:**
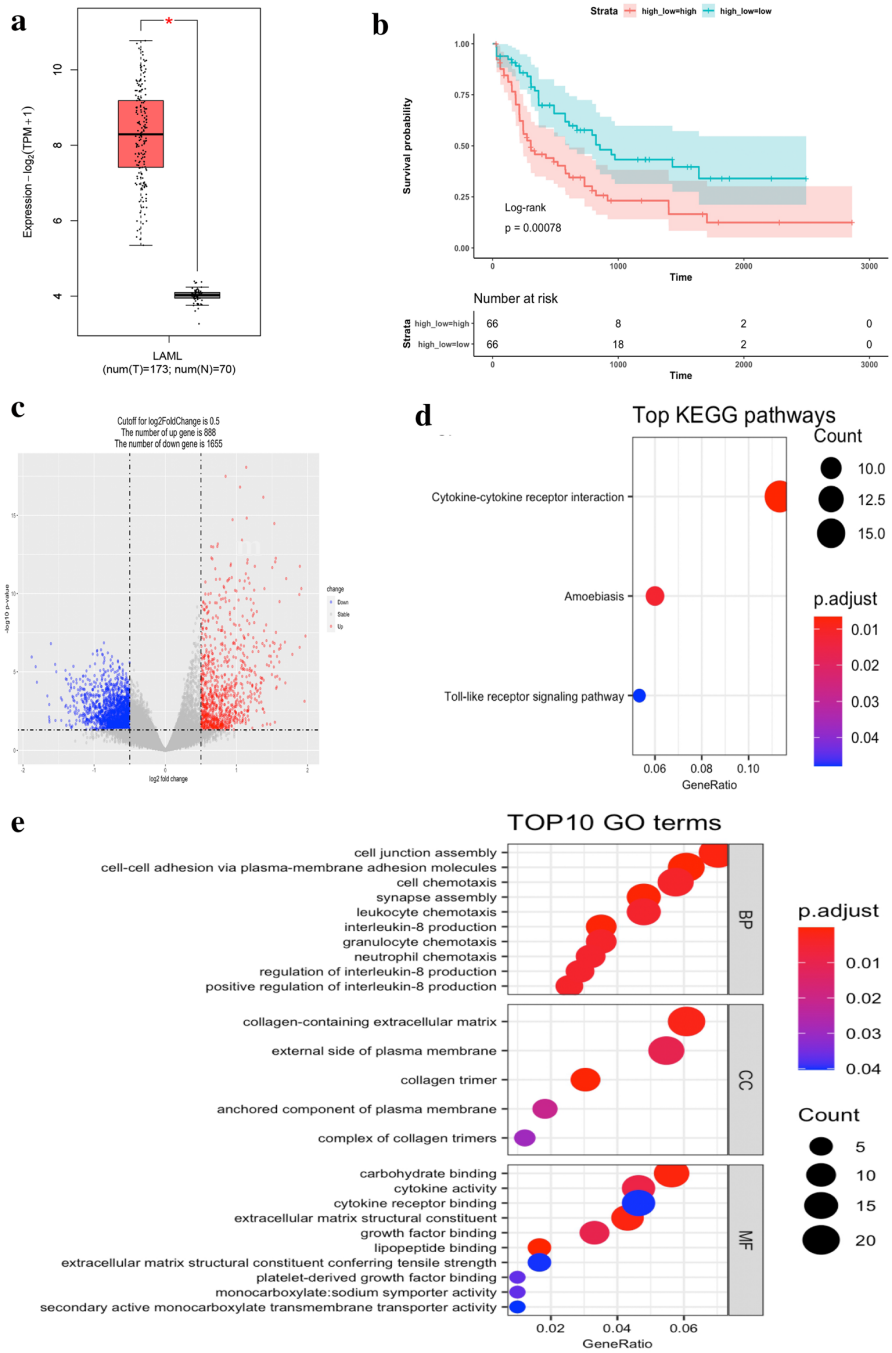
S100A4 is a risk factor for AML and is manly associated with cell adherence and cytokine regulation. **a** Expression level of S100A4 in AML in TCGA database. **b** KM plot of groups based on expression level of S100A4. **c** Differentially expressed genes between AML and normal tissues. **d** Top GO terms enriched. Color indicates the adjusted p-value, size of the sphere indicates the number of genes enriched in each pathway. **e** Top KEGG pathways enriched. Color indicates the adjusted p-value, size of the sphere indicates the number of genes enriched in each pathway

To preliminarily assess the potential importance of *S100A4* in AML, we conducted a bioinformatic analysis based on TCGA, a public database. *S100A4* was highly expressed in AML compared to normal tissues (P < 0.05) (Fig. 6a) in the GEPIA2 database. We divided patients into two groups based on their mean value of *S100A4* expression, and a KM curve showed that high expression in patients was significantly associated with poor prognosis (P = 0.00078) (Fig. 6b).

To investigate the potential mechanism of *S100A4* in AML, we conducted GO and KEGG enrichment analyses based on the DEGs between the high and low *S100A4* expression groups. A total of 888 upregulated genes and 1655 downregulated genes were identified (Fig. 6c). GO enrichment showed that *S100A4* is manly involved in the process of cell adherence, for instance, in cell junction assembly, cell–cell adhesion via plasma-membrane adhesion molecules, and collagen-containing extracellular matrixes (Fig. 6d). Pathway involved in cytokine regulation were also enriched, such as cell chemotaxis, leukocyte chemotaxis, interleukin-8 production, granulocyte chemotaxis, neutrophil chemotaxis, regulation of interleukin-8 production and positive regulation of interleukin-8 production, many of which were also enriched in the KEGG analysis (Fig. 6e). Both cell adherence and cytokine regulation are intimately associated with the development of malignant tumors.

Based on these data, *S100A4* appears to have an important role in the development and progression of AML, and it may be involved in the BM-MSC-exo-mediated phenotypic changes in AML cell lines.

## Discussion

The phenomenon of BM-MSC migration to primary tumor lesions has been thoroughly explored and is considered to be extensively involved in the tumor microenvironment [43, 44]. BM-MSC-exos have been shown to possess multiple functions and have been explored in various fields, such as cardiac repair [45], necrotizing enterocolitis [46], osteoarthritis [47], breast cancer [48], multiple myeloma [12] and leukemia [38–42]. However, the function and mechanism of BM-MSC-exos in leukemia cells are still largely unexplored. In this research, BM-MSCs were purified and cultured. Exosomes were isolated from BM-MSCs and used to explore their influence on AML cells. We found that leukemia cell lines treated with BM-MSC-exos at 50 μg/ml demonstrated enhance movement, suggesting BM-MSC-exos can facilitate migration and invasion. CD34 and CD123, a stem cell marker, were chosen as the markers for LSCs. We assessed the expression level of CD34 on leukemia cells treated with PBS or BM-MSC-exos by FCM and found that BM-MSC-exos increased the population of LSCs. A colony forming assay was performed to further confirm the effect of BM-MSC-exos on promoting proliferation in leukemia cells. Correspondingly, the transcript-level expressions of stem cell-associated genes, including *OCT4*, *KLF4*, *BMI1*, *NANOG* and *SOX2*, were significantly increased in leukemia cells upon treatment with exosomes from BM-MSCs. However, a study showed that BM-MSC-exos not only suppressed cell proliferation and cell cycle progression, but also promoted cell apoptosis in KG-1a cells [40]. Similarly, Zhang et al. [41] showed that BM-MSC-exos inhibited cell proliferation via *miR-222-3p* and promoted cell apoptosis by targeting IRF2 and negatively regulating IRF2/INPP4B signaling in THP-1 cells. In contrast to those studies, we showed that BM-MSC-exos can promote cell growth and resistance to Ara-C. We think the difference maybe caused by heterogeneity of exosomes and different AML cell lines. Altogether, these results suggest that exosomes from BM-MSCs play a key role in sustaining cell stemness and promoting cell invasion and drug resistance.

To explore the underlying mechanism of this process, we looked to S100A4, which has been reported to be a critical regulator of the cell cycle, differentiation and invasion in many kinds of cancer cells [25–30]. The relationship between BM-MSC-exos and *S100A4* regulation in AML may provide insight into the mechanisms of AML progression and recurrence. To our knowledge, this is the first study on the ability of BM-MSC-exos to increase *S100A4*. In this study, treatment of AML cells with BM-MSC-exos led to increased expression of S100A4. Further studies are warranted to determine the mechanism by which knockdown of *S100A4* reduced proliferation, invasion, and chemoresistance of leukemia cells, and rescued the phenotypes induced by treatment with BM-MSC-exos. Emerging evidence suggests that *S100A4* is overexpressed in leukemia and is associated with a poor prognosis [32, 33], This is consistent with the results of our study. Moreover, GO and KEGG enrichment analyses showed that *S100A4* is manly involved in cell adherence and cytokine regulation, which are intimately associated with malignant features. Our results suggest that BM-MSC-exos influence the proliferation, invasion and chemoresistance of leukemia cells via the upregulation of *S100A4.*

## Conclusion

In summary, our research demonstrated the role of BM-MSCs in promoting AML cell proliferation, invasion and chemoresistance via BM-MSC-exos. We found that BM-MSC-exos upregulated the expression of S100A4 of AML cells, and S100A4 acted as a key regulator of extracellular BM-MSC-exo signaling. Silencing of *S100A4* could reduce the invasion and chemoresistance of AML cells after treatment with BM-MSC-exos. The pleiotropic effects of BM-MSC-exo-S100A4 signaling in proliferation, invasion and chemoresistance suggest that S100A4 could be an effective target for AML therapy.

## Data Availability

Not applicable.

## Abbreviations

BM-MSCs: Bone marrow mesenchymal stem cells
BM-MSC-exos: Exosomes derived from bone marrow mesenchymal stem cells
FCM: Flow cytometry
CCK-8: Cell counting kit-8
qPCR: Real-time quantitative polymerase chain reaction
AML: Acute myeloid leukemia
LSCs: Leukemic stem cells
HSCs: Hematopoietic stem cells
CSCs: Cancer stem cells
PBS: Phosphate-buffered saline
FBS: Fetal bovine serum
GO: Gene ontology
KEGG: Kyoto encyclopedia of genes and genomes
Ara-C: Cytarabine
